# Surgical and Functional Outcome after Resection of 64 Petroclival Meningiomas

**DOI:** 10.3390/cancers14184517

**Published:** 2022-09-17

**Authors:** Arthur Wagner, Marie Alraun, Victoria Kahlig, Anne-Sophie Dorier, Amir Kaywan Aftahy, Denise Bernhardt, Stephanie E. Combs, Jens Gempt, Ehab Shiban, Bernhard Meyer, Chiara Negwer

**Affiliations:** 1 Department of Neurosurgery, Klinikum Rechts der Isar, Technical University Munich School of Medicine, 81675 Munich, Germany; 2 Department of Radiation Oncology, Klinikum Rechts der Isar, Technical University Munich School of Medicine, 81675 Munich, Germany; 3 Deutsches Konsortium Für Translationale Krebsforschung (DKTK), Partner Sites Munich, 80333 Munich, Germany; 4 Institute of Radiation Medicine (IRM), Department of Radiation Sciences (DRS), Helmholtz Zentrum München (HMGU), 85764 Neuherberg, Germany; 5 Department of Neurosurgery, Universitätsklinikum Augsburg, 86156 Augsburg, Germany

**Keywords:** petroclival meningioma, outcome, resection, cranial nerve, cranial neuropathy

## Abstract

**Simple Summary:**

Meningiomas are common brain tumors, which are generally treated by surgery or radiation. When these tumors are situated in difficult to reach locations within the skull, such as petroclival meningiomas, surgery may elicit impairments of cranial nerve function postoperatively. These may have a substantial impact on the postoperative quality of life of patients. Modern surgical strategies accept incomplete tumor removal in favor of preservation of cranial nerve function. With our study, we investigated the occurrence of these impairments and their recovery over time in a series of surgically treated petroclival meningiomas, applying different surgical approaches and postoperative radiation therapy in some patients. We were able to find favorable recovery rates of most postoperative impairments with a high rate of complete tumor removal. Preoperatively, we identified tumors unsuitable for complete removal due to their infiltration of the cavernous sinus or brain stem affection on magnetic resonance imaging.

**Abstract:**

**Objective:** The management of petroclival meningiomas (PCMs) remains notoriously difficult due to their close association with neurovascular structures and their complex anatomy, hence the surgical paradigm change from radical to functional resection in the past. With this study, we aimed to analyze surgical and functional outcomes of a modern consecutive series of patients with PCMs. **Methods:** We reviewed patient charts and imaging data of 64 consecutive patients from 2006 to 2018 with a PCM resected at our institution and compared surgical and functional outcomes between subgroups stratified by surgical approach. **Results:** Females comprised 67.2% of patients (*n* = 43), with a mean age of 55 years (median 56; range 21–84). Follow-up data were available for 68.8% and reached a mean of 42.3 months (range 1–129) with a median of 28.5 months. The mean tumor diameter was 37.3 mm (standard deviation (SD) 15.4; median 37.0). Infiltration of the cavernous sinus was observed in 34 cases (53.1%), and the lesions affected the brain stem in 28 cases (43.8%). Preoperative cranial nerve palsy was observed in 73.4% of cases; trigeminal neuropathy (42.2%), hearing loss (32.8%), and impairment of vision (18.8%) were the most common. A retrosigmoid approach was employed in 47 cases (78.1%), pterional in 10 (15.6%), combined petrosal in 2 (3.1%), and transnasal and subtemporal in 1 (1.6%). Fifteen cases (23.4%) were resected in a two-staged fashion. Gross total resection (GTR) was attempted in 30 (46.9%) cases without cavernous sinus infiltration and was achieved in 21 (70.0%) of these cases. Surgical complications occurred in 13 cases (20.3%), most commonly meningitis (*n* = 4; 6.3%). Postoperatively, 56 patients (87.5%) developed new cranial nerve palsy, of which 36 (63.6%) had improved or resolved on last follow up. Achieving GTR was not significantly associated with higher rates of surgical complications (chi-square; *p* = 0.288) or postoperative cranial nerve palsy (chi-square; *p* = 0.842). Of all cases, 20 (31.3%) underwent postoperative radiation. Tumor progression was observed in 10 patients (15.9%) after a mean 102 months (median 124). **Conclusions:** Surgical resection remains the mainstay of treatment for PCMs, with perioperative cranial neuropathies exhibiting favorable recovery rates. Most essentially, the preselection of patients with hallmarks of brain stem affection and cavernous sinus infiltration should dictate whether to strive for a functionally oriented strategy in favor of radical resection.

## 1. Introduction

Meningiomas continue to hold significant epidemiological significance belonging to the most common primary intracranial neoplasms [1,2,3]. Their generally high prevalence is accompanied by the considerable variability of locations from which they may arise, which dictate treatment strategies and surgical technique. A notoriously challenging and rare subgroup, petroclival meningiomas (PCMs) demand surgical control of the neighboring brain stem, cranial nerves, vessels, and skull base anatomy through a usually confined operating corridor. Modern surgical strategy has thus been geared toward tailored resection to preserve vital functionality and thus health-related quality of life (QOL) for patients, while paving the way for adjunct radiation for long-term tumor control [4,5,6,7,8,9]. Within the treatment regimen, resection invariably occupies the first step and should be striven for in any given scenario without compromising patients’ functional capacity.

It has been of academic interest to refine surgical outcomes with respect to perioperative morbidity, neurological impairment, and tumor control. In this study, we report surgical, functional, and oncological outcomes of a consecutive patient cohort with PCMs treated surgically at our institution. We aimed to condense the current standards of practice applied in a clinical setting of a modern neurosurgical unit with skull base expertise into a streamlined treatment strategy, and report on the outcome with emphasis on functional capacity of cranial neuropathies. The postoperative functional morbidity remains integral to the patients’ health-related QOL, while data on it appear lackluster in the available literature.

## 2. Material and Methods

### 2.1. Patient and Tumor Characteristics

**Figure 1:**
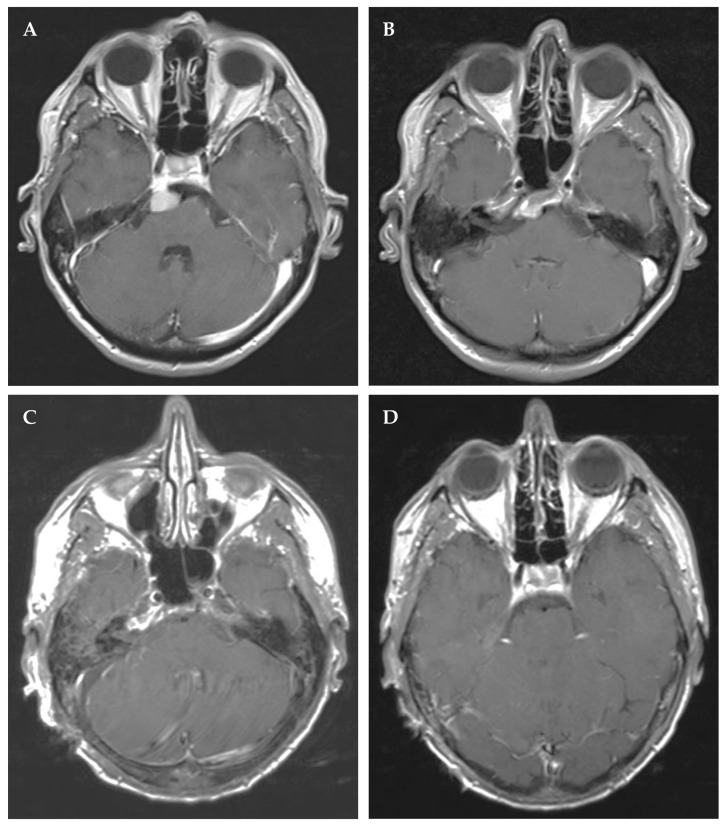
Right petroclival meningioma in a 58-year-old male with tinnitus. The small tumor had been detected 3 years prior to surgery, at an external facility. Preoperative contrast-enhanced T1 magnetic resonance imaging (MRI) in axial (**A**,**B**) reconstructions without affection of the brain stem or invasion into the cavernous sinus. Postoperative imaging after complete Simpson I resection via a retrosigmoid approach (**C**,**D**) without new postoperative deficits.

**Figure 2:**
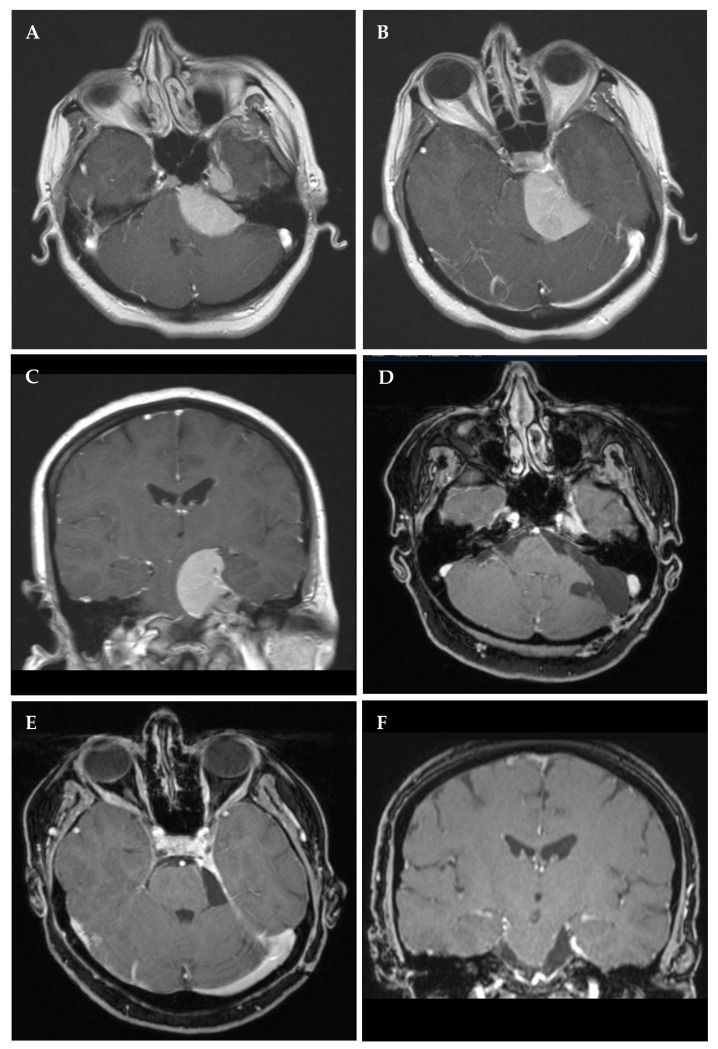
Left petroclival meningioma in a 48-year-old female with headaches and intermittent dysphagia. Preoperative contrast-enhanced T1 magnetic resonance imaging (MRI) in axial (**A**,**B**) and coronal I (**C**) reconstructions with compression of the brain stem but without invasion of the cavernous sinus. Most of the lesion was situated in the infratentorial compartment and was reached by a retrosigmoid approach, with only minor residual tumor appreciated in Meckel’s cave (**D**–**F**). Six years later, the patient underwent radiation therapy (54 Gy) for tumor progression, while she remained without deficits throughout follow-up.

**Figure 3:**
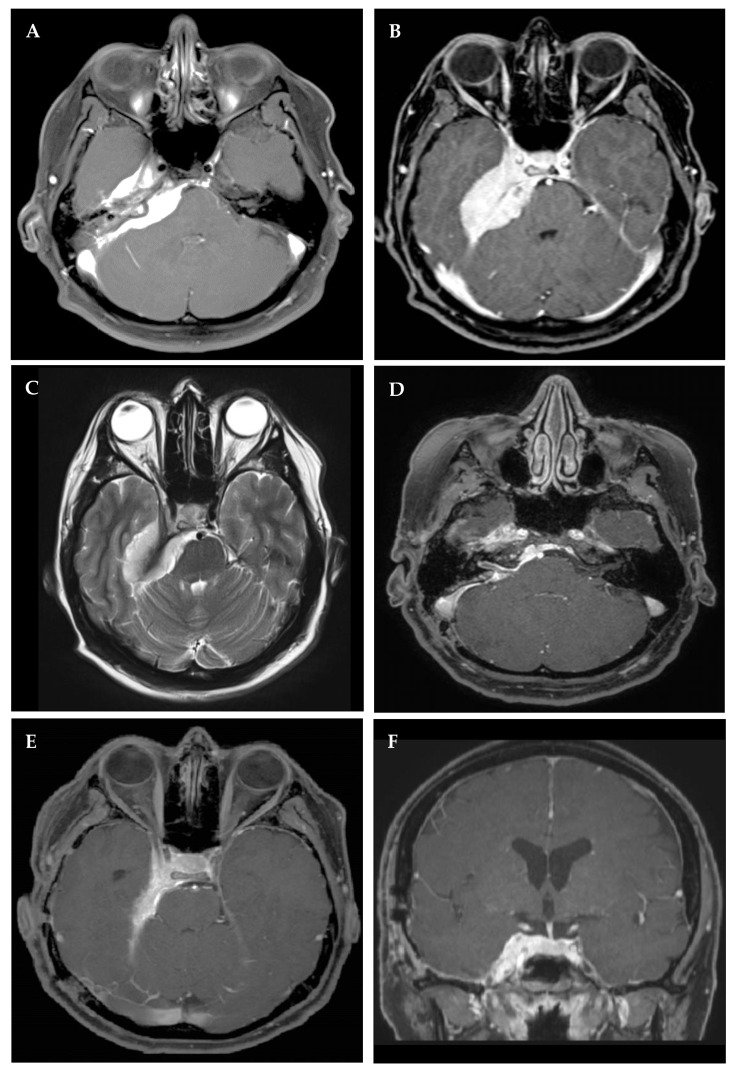
Right petroclival meningioma in a 48-year-old female who presented with tinnitus, diplopia and vertigo, undergoing a staged resection by (1) retrosigmoid and (2) pterional craniotomy. Preoperative magnetic resonance imaging (MRI) in axial contrast-enhanced T1 (**A**,**B**) and T2 (**C**) I sequences demonstrating compression of the brain stem and invasion of the cavernous sinus. Residual tumor within cavernous sinus and tentorial edge on postoperative MRI (**D**–**F**). A transient palsy of the 4th nerve was noted after the second procedure, resolving fully on follow-up.

**Figure 4:**
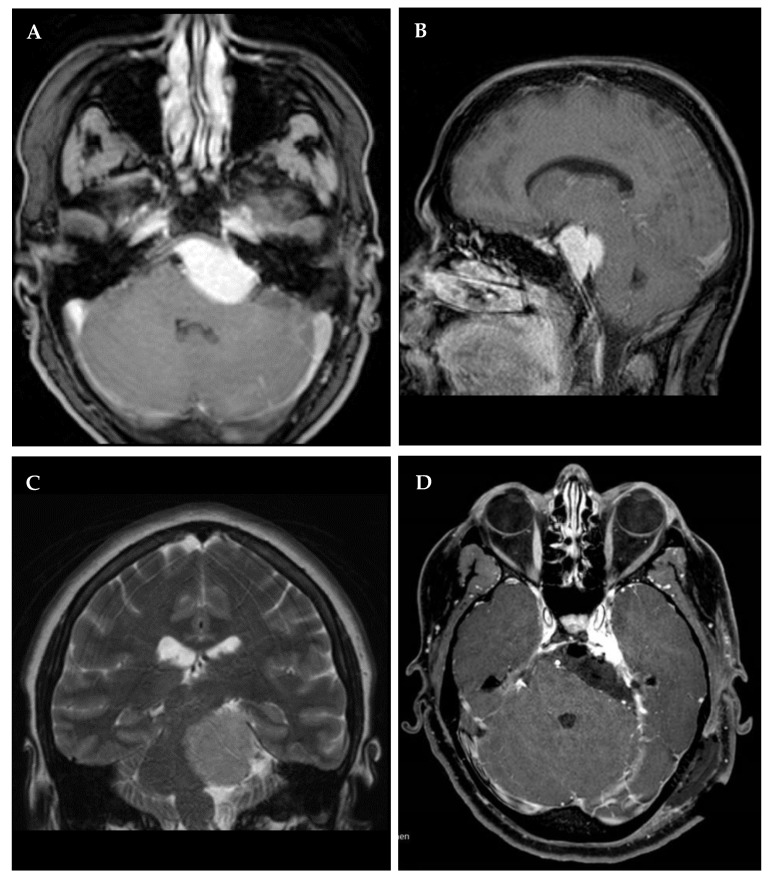
Left petroclival meningioma in a 31-year-old female who presented with trigeminal hypesthesia. Preoperative contrast-enhanced T1 magnetic resonance imaging (MRI) in axial (**A**) and sagittal (**B**) reconstructions as well as coronal T2 (**C**). Postoperative T1 MRI in axial reconstructions (**D**) after Simpson grade IV resection for decompression of the brain stem via a retrosigmoid approach.

We reviewed demographics, imaging data, surgical reports, and follow-up consultations of a consecutive cohort of patients who received PCM operations at our institution. The definition encompassed lesions located in the upper two-thirds of the clivus and medial to the 5th cranial nerve as appraised on T1 contrast-enhanced as well as T2 magnetic resonance imaging (MRI), which is in accordance with pertinent literature (Figure 1, Figure 2, Figure 3 and Figure 4) [10,11,12]. Computed tomography (CT) allowed for complementary evaluation of lesional extension into the osseous skull base. With the *BrainLab Elements*™ (BrainLab AG, Munich, Germany) software, tumor diameters were measured in millimeters in multiplanar reconstructions of pre- and postoperative imaging data.

### 2.2. Surgical Approaches and Strategy

The individual surgical approach was chosen considering various characteristics of tumor growth, such as extension into the cerebellopontine angle (CPA), the cavernous sinus, and superiorly into the middle fossa. The choice of approach generally addressed the affected skull base compartments: tumor situated within the CPA and medial posterior skull base was approached via a *retrosigmoid craniotomy*, while the tumor extensions into the middle fossa were approached via a *pterional craniotomy*. In cases with tumor extension into both the middle and posterior skull base compartments, we opted for a combination of approaches through a two-staged resection.

For the retrosigmoid craniotomy, the patient’s head was rotated 90 degrees to the contralateral side and the ipsilateral shoulder supported on a soft pad in the supine position. A curvilinear skin incision was made behind the ear and a bone flap prepared with orientation to characteristic landmarks indicating the sigmoid sinus.

For the pterional craniotomy, the supine patient’s head was rotated 45 degrees contralaterally. Here, a myocutaneous flap was prepared over the temporal bone and the craniotomy placed over the Sylvian fissure, exposing both the frontal and temporal lobe.

For selected cases with intrameatal and petrosal expansion, the *combined petrosal approach* merged an anterior petrosectomy with a retrolabyrinthine mastoidectomy for exposure of the infratentorial and supratentorial compartments by incision of the tentorium. The approach had historically been developed to provide wide access to lesions at the lateral clivus and ventrolateral brain stem, such as PCMs [13,14,15].

The few lesions predominantly at or within the clivus without extension beyond the midline, i.e., into the CPA, were resected by a *transnasal endoscopic approach*. Depending on the location of the PCM in the cranio-caudal direction, the anterior wall of the pneumatized sphenoid sinus was drilled to gain access to the superior and middle portions of the clivus, and then the PCM was addressed anteriorly.

The extent of resection was graded according to the Simpson classification (Supplementary Table S1) [16], and histopathological grading was conducted according to the World Health Organization (WHO) classification update of 2021 [16,17]. Following the principles of a functionally oriented resection, lesions infiltrating into the cavernous sinus on preoperative T1 contrast-enhanced MRI and brain stem affection on preoperative T2 MRI were not attempted for gross total resection (GTR), which was defined as Simpson grades I and II. These lesions were debulked to the maximum degree that facilitated safe adjunct radiation therapy without functional compromise; the choice of approach was governed by the same considerations as described above. These cases were labeled subtotal resections (STR) by definition, corresponding to Simpson grades III–V.

There has been considerable heterogeneity in the evaluation of disease progression for meningiomas in general, without truly reproducible radiographic criteria [18]. Some studies adjust existing response criteria of high-grade gliomas, which is currently not backed by evidence; however, the Revised Assessment in Neuro-Oncology (RANO) working group has established benchmarks for progression-free survival rates and is working on a consensus of radiographic disease progression criteria [19,20]. For the purposes of this study, any new contrast-enhancing solid mass in T1 MRI sequences on follow-up was defined as tumor progression, which presupposed treatment decisions.

Every individual case was discussed in an interdisciplinary neurooncological board meeting constituting specialists in neurosurgery, radiooncology, neurooncology and neuroradiology with expertise in skull base pathologies. Generally, the consensus foresaw salvage RT in patients with CNS WHO 1 meningiomas with residual significant tumor burden or disease progression on MRI, and early adjuvant radiation therapy (RT) in CNS WHO 2 meningiomas after STR, which is recommended by current treatment guidelines [21].

### 2.3. Functional Outcome

Pre- and postoperative assessments of neurological function and distinct examinations of cranial nerve function represented standard care for all patients. In cases with preoperatively apparent cranial nerve palsies, an extended work-up was added appropriately. Loss of visual acuity or visual field deficits were examined by standardized ophthalmological examination with Snellen charts and Goldmann perimetry, whereas hearing impairment was graded by pure tone audiometry. Facial nerve palsy was graded according to House & Brackmann [22].

### 2.4. Ethical Declaration and Statistical Analysis

All procedures performed in studies involving human participants were in accordance with the ethical standards of the institutional and national research committee as well as the 1964 Helsinki declaration and its later amendments or comparable ethical standards. The study group acquired approval by the local ethics committee; the requirement to obtain informed consent was waived (Ethikkommission der Technischen Universität München, registration no. 5551/12).

We used IBM SPSS version 25.0 (IBM corp., Armonk, NY, USA) [23] with *t* tests and chi-square testing for parametric and non-parametric comparisons of means and proportions, respectively, and the level of significance was defined a priori as *α* = 0.05.

## 3. Results

### 3.1. Cohort and Lesion Characteristics

Sixty-four patients underwent resections of PCMs between 2006 and 2018. Baseline characteristics are listed in Table 1. Of 64 patients with a mean age of 55 years (median 56; range 21–84), 67.2% were female. The majority of tumors were classified as central nervous system (CNS) WHO grade 1 (95.3%) with an average largest diameter of 37.3 mm (standard deviation (SD) 15.4; median 37.0). Table 2 lists the relations of PCMs to neighboring neurovascular structures as determined by preoperative imaging, with or without corresponding symptoms. Among the cranial nerves, the trigeminal nerve was most commonly affected on imaging (76.6% of cases). Infiltration of the cavernous sinus was observed in 53.1% of cases, and the lesion had affected the brain stem on preoperative T2 MRI in 43.8% of cases.

### 3.2. Preoperative Complaints and Functional Status

Preoperative cranial nerve palsy was observed in 47 patients (73.4%); trigeminal neuropathy (*n* = 27; 42.2%), hearing loss (*n* = 21; 32.8%), and impairment of visual acuity (*n* = 12; 18.8%) were the most common (Table 3). All four patients with preoperative VII palsy were House & Brackmann grade 3. Within the subgroups of patients with preoperative impairment of vision and hearing, three patients (4.7%) exhibited amaurosis, and two (3.1%) had surdity.

Unspecific complaints such as vertigo (*n* = 24; 37.5%), gait ataxia (*n* = 17; 26.6%), and cephalgia (*n* = 12; 18.8%) were less prevalent than specific cranial nerve palsy. Only three patients (4.7%) presented after suffering a first-time seizure event. The preoperative Karnofsky Performance Scale (KPS) scores amounted to a median 90% (range 80–100%).

### 3.3. Surgical Outcome

In 49 cases (76.6%), a single-staged resection was conducted and in 15 cases (23.4%) a combination of two different approaches over two stages (Table 4). In these cases, the second resection was conducted after a median of 12.5 days (range 1–127). The median time from cut to suture amounted to 262.5 ± 122.7 min.

A GTR was attempted in 30 of 64 cases (46.9%) and was achieved in 21 (70.0%) of these. For the remaining 34 cases (53.1% of total), the primary surgical strategy was aimed at the decompression of neural structures without violating the lateral wall of the cavernous sinus, thus constituting subtotal resections by definition.

Adjunct RT followed surgical treatment in 20 cases (31.3%) after a median 63 days (range 51–89 days). Of these, 17 (26.6%) were CNS WHO grade 1, and 3 (4.7%) were CNS WHO grade 2. During the median hospital stay of 10 days, surgical complications occurred in 13 cases (20.3%), which mostly included meningitis (*n* = 4; 6.3%) requiring antibiotic treatment, hydrocephalus (*n* = 3; 4.7%) requiring a cerebrospinal fluid (CSF) diversion procedure, CSF fistula requiring revision surgery in two cases (3.1%), and postoperative epidural hematoma requiring revision surgery as well as wound infection requiring wound debridement in two cases (3.1%) each. A GTR was not associated with a significantly higher rate of complications (GTR: 3 (4.7%) vs. STR: 10 (15.6%); chi-square: *p* = 0.538). After stratification of the cohort by the median tumor size (37.0 mm), the subgroup with PCMs below 37.0 mm in largest diameter had significantly fewer complications compared to larger PCMs (2 (3.1%) vs. 10 (15.6%); chi-square: *p* = 0.015).

### 3.4. Functional and Oncological Outcome

Follow-up data were available for 44 patients (68.8%) and reached a mean 42.3 months (range 1–129) with a median 28.5 months.

**Figure 5:**
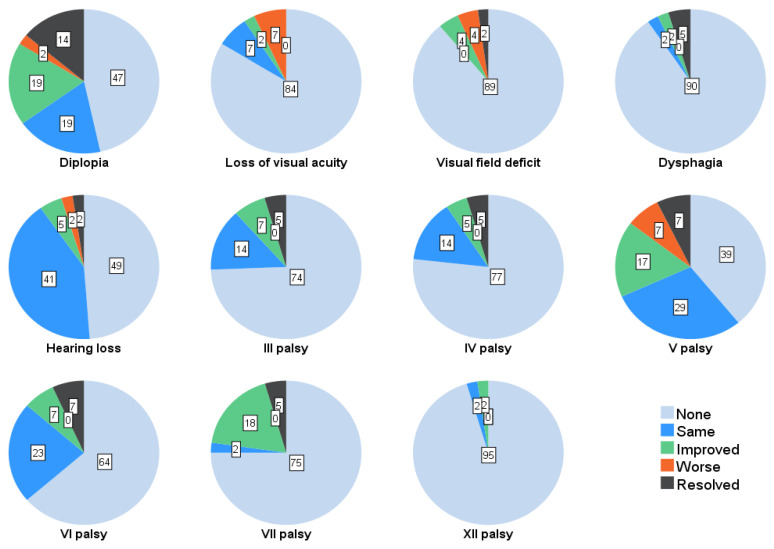
Proportions (%) of all patients with complaints and cranial nerve palsies on last follow up compared to status at discharge.

Postoperatively, 56 patients (87.5%) had cranial nerve palsy, of which 41 patients (63.6%) had improved or resolved on last follow up (Figure 5). Trigeminal neuropathy had the highest resolution rate with 16.6% (*n* = 11) as assessed through clinical examination, while visual impairment only improved in one patient (1.6%) on ophthalmological examination and hearing loss in three patients (4.7%) in pure tone audiometry. Of 15 patients (23.4%) with a postoperative facial nerve palsy, seven (10.9%) were grade IV, four (6.3%) grade III, three (4.7%) grade II and one (1.6%) grade V.

Improvement was noted in five patients (7.8%) from grade IV to III, two patients from grade IV to II (3.1%), four patients (6.3%) from grade III to II, and in all three grade II patients (4.7%) to resolution. Diplopia, even in the absence of a clear cranial nerve palsy related to oculomotion, improved in 12 patients (18.6%) and resolved in 9 patients (14.0%), as assessed on clinical examination. The postoperative median KPS remained at 90% on follow-up without a significant difference between subgroups of GTR and STR (90% each; chi-square: *p* = 0.690). Tumor progression was observed in 10 patients (15.9%); the mean time to tumor progression was 102.4 months (95% confidence interval (CI), 85.7–119.0) as calculated by estimated survival analysis. The median time amounted to 124 months (95% CI, 64.8–183.2). After stratification into subgroups of GTR and STR, no significant benefit for the former was noted (106.3 versus 85.5 months; Student’s *t* test: *p* = 0.676). In the subgroup of patients with STR (*n* = 45; 70.3% of total), follow-up was available in 41 cases (64.3%) with a mean time of 40.4 months. No significant difference was found between mean times of tumor recurrence for patients undergoing additive radiation (114.3 months; 95% CI, 91.8–136.9) and those with a watchful waiting regimen (92 months; 95% CI 67.2–116.8; Student’s *t* test: *p* = 0.460).

Linear regression analyses revealed that GTR was not significantly associated with higher rates of surgical complications (*p* = 0.288) or postoperative cranial nerve palsy (*p* = 0.842).

## 4. Discussion

### 4.1. Surgery

The safe removal of PCMs first and foremost constitutes a technical challenge related to their intrinsic anatomic complexity and delicate topographic relation to vital midline structures, vessels, and cranial nerves. It comes as no surprise that these entities had historically been deemed inoperable; only with the advent of microneurosurgery were genuine attempts aiming for full resection made [9,14,24,25,26,27]. Radical resection was traditionally advocated for in favor of deliberate subtotal and functionally preserving removal [26,28,29]. With this paradigm came complications, mostly constituting cranial neuropathies and often including a sacrifice of hearing through the approach [14,25,27]. Of note, these studies report GTR rates of 70–85% and opposite complication rates of 46%, although these data are based on fairly small cohorts [24,29,30].

These principles have been abandoned over time, with the growing realization of intact cranial nerve function as paramount for postoperative health-related QOL. Some recent studies have investigated cranial nerve deficits in relation to surgical approach and their recovery rates on long-term follow-up. A meta-analysis by Di Carlo et al. concluded that the choice of surgical approach influences palsy rates only of the 4th and 7th cranial nerves [31]. They analyzed favorable recovery rates across all postoperative cranial nerve palsies, with the exception of 8th-nerve palsy persisting in 8.9% of cases on follow-up. Almefty et al. reported a cranial neuropathy rate of 89% on presentation, which persisted in 33% of cases in their relatively long follow-up observation, with an average of 72 months [11]. In line with our data, achieving GTR did not significantly impact the rate of postoperative cranial neuropathy. Nanda et al. found new postoperative cranial neuropathy in 44% of patients, a third of these being found permanent at their mean follow-up of 22 months [32]. Other studies reported a postoperative cranial nerve deterioration in 100% of cases despite GTR in only 37% [33].

In our study, GTR was achieved in 21 patients, in whom the PCM had not yet infiltrated into the cavernous sinus or affected the brain stem as appreciated on preoperative T2 MRI, and mostly through one surgical approach. Achieving GTR in these cases, however, did not significantly influence the postoperative occurrence of deficits, which may be related to the lesion’s size and thus preoperatively existing neurological dysfunction, allowing smaller tumors to be resected fully without injury to neural structures. This observation is reciprocated by two recent studies reporting excellent outcomes after resection of exclusively small PCMs [34,35]. It follows that PCMs presenting with the hallmarks of cavernous sinus or brain stem affection necessitate a wholly different management concept, rendering any attempt at GTR unfeasible and qualifying this subgroup of PCMs for adjunct radiation therapy following surgical STR [36]. Depending on the extent of resection, the patient’s functional status, and association with surrounding structures such as the brain stem at risk for RT, observation may be a viable alternative in these cases, although this is not yet convincingly supported by evidence. It seems safe to assume that PCMs’ inherent technical difficulty is impossible to eliminate but must be overcome. As such, a surgeon’s familiarity with one surgical approach gains significance, determines its applicability to a variety of scenarios, and should be favored over a “master of none” philosophy.

Naturally, the definitive surgical strategy and oncological concept must be tailored to the individual case. The infiltration of the cavernous sinus and affection of the brain stem serve as indicators for lesions best addressed by functionally oriented STR and adjunct RT.

### 4.2. Radiotherapy

In the last decades, RT and especially high-precision techniques such as fractionated radiotherapy (FRT), particle therapy or intensity-modulated radiotherapy (IMRT) have become cornerstones in the multimodal treatment of meningiomas. An FRT of 50−54 Gy (1.8–2.0 Gy per fraction) can be applied for volumes that are not suitable for single fractionation or hypofractionation. This technique offers the potential for a broader safety profile, especially for tumors involving sensitive organs at risk (OAR). Previous reports of meningioma patients treated with either FRT, particle therapy or IMRT reported progression-free survival rates of 96.6% after 5 years and 91% after 10 years. Meanwhile, QOL was unchanged in 47.7% of the patients, and 37.5% showed improvement [21,37,38,39]. Independent of technique, radiosurgery approaches such as Gamma Knife treatment are comparable with respect to progression-free survival. In selected cases of small meningiomas, stereotactic radiosurgery allowed for a single application of 12−16 Gy [39,40,41,42]. While single-fraction treatment of small lesions is both effective for controlling tumors and sparing OARs, it can be a considerable challenge to balance tumor control and radiation-induced toxicity for larger tumors, especially skull base tumors. Hypofractionated Gamma Knife Radiosurgery (e.g., 25 Gy in 5 fractions) can optimize said balance of tumor control and normal tissue toxicity in comparison to single-fraction radiosurgery [40,43,44,45].

Issues remain on early adjuvant or additive RT versus a wait-and-see strategy. Some reports implicate that early treatment improves and prolongs progression-free survival when compared to surgery alone [46,47]. However, RT performed as salvage-treatment after surgery is comparably effective and therefore might be withheld to minimize side effects and spare patients from additional treatment that might not be necessary [37,38]. Surgery usually aims for a GTR, but the multimodal approach of STR followed by RT is associated with disease control and survival rates similar to those reported for GTR [39,40,48,49]. Alternatively, an elective combined resection followed by irradiation of residual tumors provides a planned approach for the tumor and balances tumor control and toxicity [21,39,40]. In the case of skull base meningioma, multimodal concepts combining STR and RT are guideline recommended to minimize toxicities. Multidisciplinary diagnoses, treatment decisions and therapy as planned in neurooncological tumor boards remain the recommended standard of care [21].

## 5. Study Limitations

Most apparently, the results of this study are somewhat diminished by its retrospective design and some lacking follow-up data. This most likely affects the statistical indifference in oncological outcomes between patients undergoing adjunct radiation and those without.

Moreover, we opted to provide only a gross estimate of tumor size via the largest diameter in lieu of a succinct volumetric analysis; we hold that our principal surgical strategy foresaw not the “volumetric” reduction of tumor mass but rather a functional resection as demonstrated by the data and in accordance with modern paradigms.

Still, we were able to collect a sizable cohort with a moderate follow-up duration and thorough data on neurological function as well as tumor recurrence on imaging.

## 6. Conclusions

Surgical resection remains the mainstay of treatment for PCMs, with perioperative cranial neuropathies exhibiting favorable recovery rates. Most importantly, the preselection of patients with hallmarks of brain stem affection and cavernous sinus infiltration should dictate whether to strive for a functionally oriented strategy rather than a radical resection.

## Figures and Tables

**Table 1:** Baseline characteristics of study cohort. N, number; mm, millimeters; CNS WHO, Central Nervous System World Health Organization grading system.

N		64	
**Mean age at surgery (median; range)**		**55** **(56; 21–84)**	
**Female sex (n; %)**		43 (67.2%)	
**Mean preoperative tumor diameter in mm (median; range)**		37.3 (37.0; 13–92)	
**Localization (*n*; %)**	**left**	29	45.3%
	**right**	35	54.7%
**CNS WHO grade**	**1**	61	95.3%
	**2**	3	4.7%

**Table 2:** Numbers of cases with affiliation of PCM with surrounding structures on preoperative imaging, with or without corresponding symptoms. * determined on preoperative T2 MRI. N, number; %, percentage of entire cohort (64).

Affection of Cranial Nerves on Imaging	N	%
II	16	25.0
III	20	31.3
IV	33	51.6
V	49	76.6
VI	37	57.8
VII	34	53.1
VIII	33	51.6
IX	12	18.8
X	8	12.5
XI	8	12.5
XII	3	4.7
**Infiltration of**		
Internal auditory canal	27	42.2
Cavernous sinus	34	53.1
Meckel’s cave	35	54.7
Nasal sinus	2	3.1
Orbit	7	10.9
Sellar region	17	26.6
**Compression of third ventricle**	10	15.6
Brain stem affection *	28	43.8
**Osteolysis of skull base**	11	17.2

**Table 3:** Numbers of cases with preoperative subjective complaints and cranial nerve palsies. N, number; %, percentage of entire cohort (64).

Complaint	N	%
Diplopia	16	25.0
Loss of visual acuity	12	18.8
Visual field deficit	6	9.4
Dysphagia	3	6.3
Hearing loss	19	32.8
**Cranial nerve palsy**		
III	9	14.1
IV	5	7.8
V	27	42.2
VI	13	20.3
VII	4	6.3
XII	1	1.6

**Table 4:** Surgical approaches for single (*n* = 49) and staged (*n* = 15) resections. * 2nd pterional † 2nd retrosigmoidal.

	Single-Staged (*n* = 49)	Two Stages (*n* = 15)
Surgical Approach (N; %)		
Retrosigmoidal	42 (85.7)	9 (60.0) *
Pterional	6 (12.2)	4 (26.7) †
Transnasal Transsphenoidal	1 (2.0)	-
Combined Petrosal	-	2 (13.3) *

## Data Availability

The data presented herein is available from the corresponding author on reasonable request.

## Supplementary Materials

The following supporting information can be downloaded at: https://www.mdpi.com/article/10.3390/cancers14184517/s1, Table S1: Simpson Grading System.
