# Supplementary material for: Surgical and Functional Outcome after Resection of 64 Petroclival Meningiomas
Source: Cancers (Basel). 2022 Sep 17;14(18):4517. doi: 10.3390/cancers14184517 (PMC9496694; doi:10.3390/cancers14184517)
Supplement: Supplementary file 1 [file cancers-14-04517-s001.zip › cancers-1901850-supplementary.pdf]

cancers

MDPI

Supplementary

Surgical and functional outcome after resection of 64 petro-clival meningiomas

Table S1. Simpson Grading System.

**Macroscopically complete removal of tumour, with excision of its dural attachment, and of any abnormal bone. Includes resection of venous sinus if involved.**	**I**
Macroscopically complete removal of tumour and its visible extensions with coagulation of its dural attachment.	II
Macroscopically complete removal of the intradural tumour, without resection or coagulation of its dural attachment or its extradural extensions.	III
Partial removal, leaving intradural tumour in situ.	IV
Simple decompression, with or without biopsy	V
